# Hyperoxia Extends Time to Exhaustion During High-Intensity Intermittent Exercise: a Randomized, Crossover Study in Male Cyclists

**DOI:** 10.1186/s40798-016-0059-7

**Published:** 2016-08-24

**Authors:** Toshiyuki Ohya, Ryo Yamanaka, Hayato Ohnuma, Masahiro Hagiwara, Yasuhiro Suzuki

**Affiliations:** Department of Sports Science, Japan Institute of Sports Sciences, Tokyo, 115-0056 Japan

**Keywords:** Hyperoxic, Arterial oxygen saturation, Repeated-sprint, Training, Exercise-induced arterial hypoxemia

## Abstract

**Background:**

Some endurance athletes exhibit exercise-induced arterial hypoxemia during high-intensity exercise. Inhalation of hyperoxic gas during exercise has been shown to counteract this exercise-associated reduction in hemoglobin oxygen saturation (S_a_O_2_), but the effects of hyperoxic gas inhalation on performance and S_a_O_2_ during high-intensity intermittent exercise remain unclear. This study investigated the effects of hyperoxic gas inhalation on performance and S_a_O_2_ during high-intensity intermittent cycling exercise.

**Methods:**

Eight male cyclists performed identical intermittent exercise tests (five sets of 3-min high-intensity cycling alternated with 3-min active recovery periods) under two different inspired air conditions, hyperoxia (HO; FiO_2_ = 0.36) and normoxia (NO; FiO_2_ = 0.21). The fifth set of each test was terminated at exhaustion, and the exercise time to exhaustion was recorded. Variables associated with arterial oxygen saturation (SpO_2_) were measured using an ear pulse oximeter.

**Results:**

Time to exhaustion under HO conditions was significantly longer than under NO conditions (34.9 ± 4.6 vs. 30.0 ± 2.5 min, *P* = 0.004, ES = 1.32). SpO_2_ was maintained under HO conditions but decreased under NO conditions.

**Conclusions:**

Hyperoxic gas inhalation during the entire high-intensity intermittent exercise enhanced exercise performance in male cyclists.

## Key Points

• SpO_2_ was maintained under hyperoxia conditions, while it decreased under normoxia conditions.

• A strong correlation exists between the delta of SpO_2_ and the delta of endurance time to exhaustion in participants who exhibit exercise-induced arterial hypoxemia during the intermittent cycling exercise tests under normoxic conditions.

• Hyperoxic gas inhalation during the entire high-intensity intermittent exercise enhanced exercise performance in male cyclists.

## Background

High-intensity intermittent exercises are frequently used in training programs to improve physiological responses, such as maximizing the activities of mitochondrial enzymes [14], reducing glycogen utilization and lactate accumulation during matched-work exercise [3, 5, 12], and improving sports performance [8, 9]. These exercises, which maximize workload, result in greater training adaptations and performance improvements than less strenuous exercises. High-intensity intermittent exercises often reduce hemoglobin oxygen saturation (S_a_O_2_) and increase tissue hypoxia [7, 21], reducing the amount of oxygen available to the muscles during exercise. Around 50 % of highly trained athletes exhibit exercise-induced arterial hypoxemia [25]. Preventing the reduction in S_a_O_2_ during high-intensity intermittent exercise might contribute to improved performance and maintenance of workload.

Inhalation of hyperoxic gas during exercise has been shown to counteract the reduction in S_a_O_2_ that occurs during high-intensity intermittent exercise [21, 22, 30]. Nummela et al. [21] reported that inhalation of hyperoxic gas (fraction of inspiratory oxygen (FiO_2_) = 0.40) during an entire session of high-intensity intermittent exercise consisting of three sets of 300 m of running at different speeds on a treadmill, prevented S_a_O_2_ levels from decreasing. Several studies have shown that exposure to hyperoxic conditions improves exercise performance during continuous exercise lasting more than 3 min [24, 27, 33]. One previous study examined the effects of inhaling hyperoxic gas during the rest periods between high-intensity intermittent exercise sessions lasting more than 3 min on performance and S_a_O_2_ [22]; this study revealed that the S_a_O_2_ recovery time was significantly shorter under hyperoxic (FiO_2_ = 0.99) than under normoxic conditions. However, there was no significant difference in exercise performance [22]. Although high-intensity intermittent exercise performance was not improved by hyperoxia in that trial [22], the participants were not exposed to hyperoxic conditions during the entire high-intensity intermittent exercise, only during the rest periods. Thus, the actual performance and SaO_2_ effects of inhaling hyperoxic gas during high-intensity intermittent exercise remain unclear.

This study investigated the effects of hyperoxic gas inhalation on performance and S_a_O_2_ during high-intensity intermittent exercise. We hypothesized that inhalation of hyperoxic gas would improve high-intensity intermittent exercise performance by maintaining S_a_O_2_. To test this hypothesis, five 3-min sets of high-intensity cycling were alternated with 3 min active recovery periods under two different inspired air conditions, hyperoxia (HO; FiO_2_ = 0.36) and normoxia (NO; FiO_2_ = 0.21). Participants were exposed to the inspired air conditions for the entire high-intensity intermittent exercise.

## Methods

### Participants

Eight male cyclists participated in this study (means ± standard deviation (SD): age, 20.4 ± 1.9 years; height, 167.0 ± 5.9 cm; body mass, 58.2 ± 8.2 kg). Their weekly cycling training typically consisted of five sessions (300 km per week). None of the participants had any history or clinical signs of cardiovascular or pulmonary disease. Written consent to participate was obtained from all participants after informing them of the purpose of the experiment, the procedure, and the possible risks. This study was approved by the Human Subjects Committee at the Japan Institute of Sports Sciences and performed in accordance with the ethical standards of the Declaration of Helsinki.

### Experimental Overview

This was a single-blind randomized control trial. Participants visited the laboratory three times. During the first visit, they performed a maximal graded exercise test. During the second and third visits, which were separated by at least 48 h, five repeated high-intensity 3-min cycling sets were alternated with 3-min periods of active recovery at 20 W on a cycle ergometer (Excalibur Sport 925900; Lode BV, Groningen, The Netherlands) under HO or NO conditions. All experimental trials were conducted at the same time of the day (±1 h) for each subject to minimize the effects of diurnal variation. Participants were instructed to grip the handlebars while cycling, and all participants were familiarized with cycling on the cycle ergometer before the study. Participants were instructed not to eat within the 3 h prior and to refrain from drinking caffeinated beverages at least 10 h prior to the test.

### Maximal Graded Exercise Test

The maximal graded exercise test was performed on the cycle ergometer to determine peak oxygen uptake ($\overset{.}{\mathrm{V}}{\mathrm{O}}_{2\mathrm{peak}}$) and maximum work rate. After a 5-min warm-up at 100 W, the power output was increased by 40 W every 3 min until exhaustion. The participants were asked to maintain a cadence of 90 rpm during this test, and the test was terminated when the pedal cadence dropped below 85 rpm for 5 s despite vigorous encouragement. The maximum work rate was defined as the power output during the last stage of the maximal graded exercise test. If a participant could not maintain the work rate during the last stage for at least 1 min, the power output of the previous stage was recorded as the maximum work rate.

### Intermittent Cycling Exercise Tests

Intermittent cycling exercise tests were conducted to assess the participants’ endurance (time to exhaustion) under different inspired air conditions. A standardized whole-body warm-up, which consisted of 5 min of stretching exercises and 5 min of cycling at 100 W, was performed prior to the intermittent cycling exercise tests. This was followed by 5 min of passive rest seated on the saddle before the testing commenced. The fractional content of oxygen in the inspired air (for HO or NO) was randomly assigned. Hyperoxic gas was inhaled from 2 min prior to the onset of the intermittent cycling exercise tests under HO conditions. The intermittent cycling exercise tests comprised five sets of 3-min high-intensity cycling separated by 3 min of active recovery (20 W) [1]. The fifth set of the test was terminated at exhaustion. In all trials, the participants were required to assume the start position 5 s before the start of the next cycling period. Handlebar and saddle height were adjusted according to the preference of the participant and were kept constant for each trial. Work rates were adjusted to correspond to 90 % of each individual’s maximum work rate determined by the maximal graded exercise test under normoxic conditions and ranged from 216 to 288 W (mean work rate 270 W). The participants were asked to maintain a cadence of 90 rpm during the test, and strong verbal encouragement was provided to each participant during all cycling. The test was terminated when the pedal cadence dropped below 85 rpm for 5 s despite vigorous encouragement.

### Measurements

#### Cardiorespiratory Measurements

Breath-by-breath respiratory gas exchange was measured using an automated analysis system (AE-310s; Minato Medical Science, Osaka, Japan) to determine ventilation ($$ \overset{.}{\mathrm{V}}\mathrm{E} $$), oxygen uptake ($$ \overset{.}{\mathrm{V}}{\mathrm{O}}_2 $$), and carbon dioxide production ($$ \overset{.}{\mathrm{V}}\mathrm{C}{\mathrm{O}}_2 $$) during the maximal graded exercise test. Respiratory gas exchange values were averaged every 30 s during the maximal graded exercise test. Heart rate (HR) (RS800; Polar Electro, Kempele, Finland) was averaged every 30 s during both the maximal graded exercise test and the intermittent cycling exercise tests. The gas analysis system was calibrated using a gas mixture of known O_2_ and CO_2_ concentrations before each test. The volume transducer was calibrated before each test using a 2-L syringe (Minato Medical Science). Peak values were recorded for the maximal graded exercise test. The $$ \overset{.}{\mathrm{V}}{\mathrm{O}}_{2\mathrm{peak}} $$ and maximal $$ \overset{.}{\mathrm{V}}\mathrm{E} $$$$ \left(\overset{.}{\mathrm{V}}{\mathrm{E}}_{\mathrm{peak}}\right) $$ were defined as the highest $$ \overset{.}{\mathrm{V}}{\mathrm{O}}_2 $$ and $$ \overset{.}{\mathrm{V}}\mathrm{E} $$ attained during the maximal graded exercise test. Arterial oxygen saturation (SpO_2_) was measured using an ear pulse oximeter (Rad-5; Mashimo, Irvine, CA, USA), sampled at 0.5 Hz during the intermittent cycling exercise tests. The SpO_2_ values from 150 to 180 s were averaged for each high-intensity cycling period. The values during the last 30 s of the fifth stage of the test were also averaged. Exercise-induced arterial hypoxemia was defined as a progressive and persistent decrease in S_a_O_2_ during exercise and a drop between resting and the end of the exercise of more than 4 % [20, 26]. Ratings of perceived exertion (RPE) were obtained using Borg’s 6–20 scale [2] during the intermittent cycling exercise tests.

#### Blood Lactate Concentration

At 30 s after the end of each high-intensity cycling period during the intermittent cycling exercise test, fingertip blood samples (0.3 μl) were collected to measure blood lactate concentrations ([La]_b_) using the enzymatic-amperometric detection method (Lactate Pro 2LT-1730; Arkray, Kyoto, Japan).

### Statistical Analysis

Data are expressed as means ± SD. Statistical analyses were performed using IBM SPSS Statistics for Windows, Version 19.0 (IBM Corp., Armonk, NY, USA). The significance of the differences in SpO_2_, RPE, HR, and [La]_b_ between HO and NO conditions during the intermittent cycling exercise tests were assessed using two-way repeated measures analysis of variance (ANOVA). The effect size (ES) as partial eta squared (*η*^2^) was determined. The significance of interactions was assessed using Bonferroni post hoc tests. Paired *t* tests were used to determine the significance of differences in endurance time to exhaustion during the intermittent cycling exercise tests. The ES, calculated as Cohen’s *d*, was determined. Correlations between the delta of SpO_2_ (the values between hyperoxia and normoxia at exhaustion) and the delta of endurance time to exhaustion (the values between hyperoxia and normoxia) were determined and tested for significance using Pearson’s product-moment test. Differences were considered significant if *P* < 0.05.

## Results

### Maximal Graded Exercise Test

Participants were able to maintain the required cadence during the maximal graded exercise test for times ranging from 13 to 21 min (mean 16 min). The mean values obtained from the maximal graded exercise test for $\overset{.}{\mathrm{V}}{\mathrm{O}}_{2\mathrm{peak}}$, $\overset{.}{\mathrm{V}}{\mathrm{E}}_{\mathrm{peak}}$, and HR_max_ were 59.1 ± 7.1 mL kg^−1^ min^−1^, 149.7 ± 11.7 L min^−1^, and 194 ± 6 beats min^−1^, respectively.

### Intermittent Cycling Exercise Tests

#### Time to Exhaustion

**Fig. 1:**
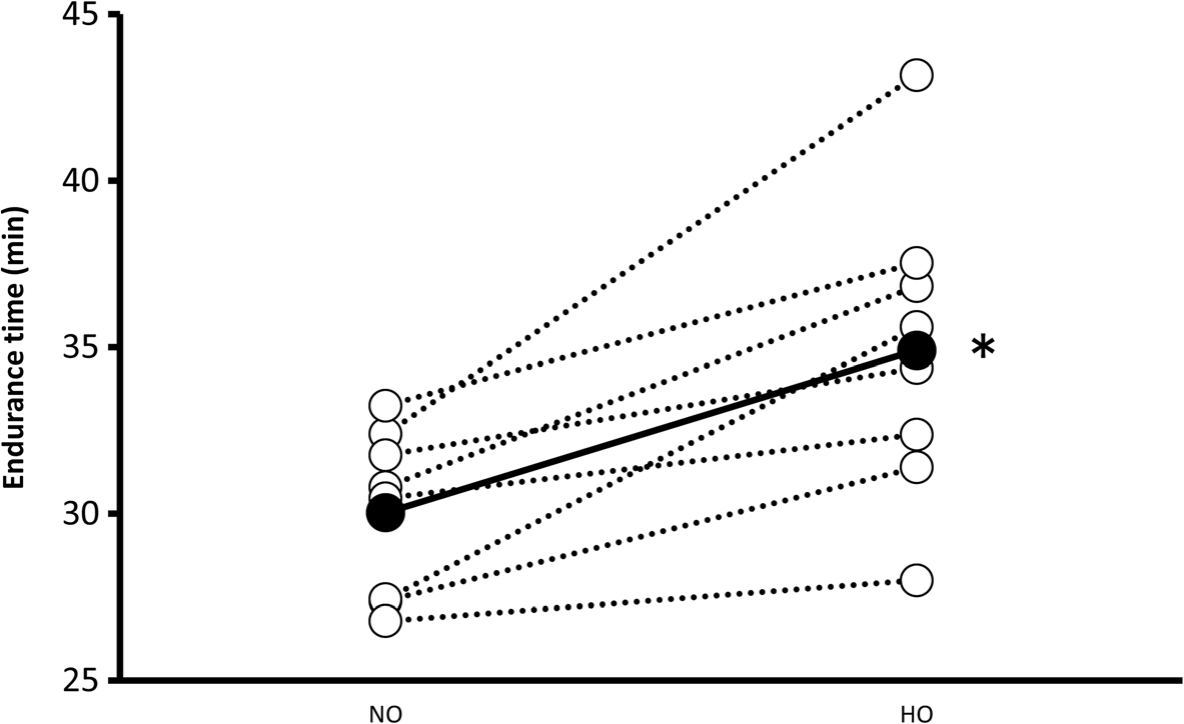
Individual and mean endurance times are shown. Hyperoxic (HO) conditions, FiO_2_ = 0.36; normoxic (NO) conditions, FiO_2_ = 0.21. Values are expressed as *white circle* = individuals and *black circle* = means (*n* = 8). *Significant difference vs. NO (*P* < 0.05)

**Fig. 2:**
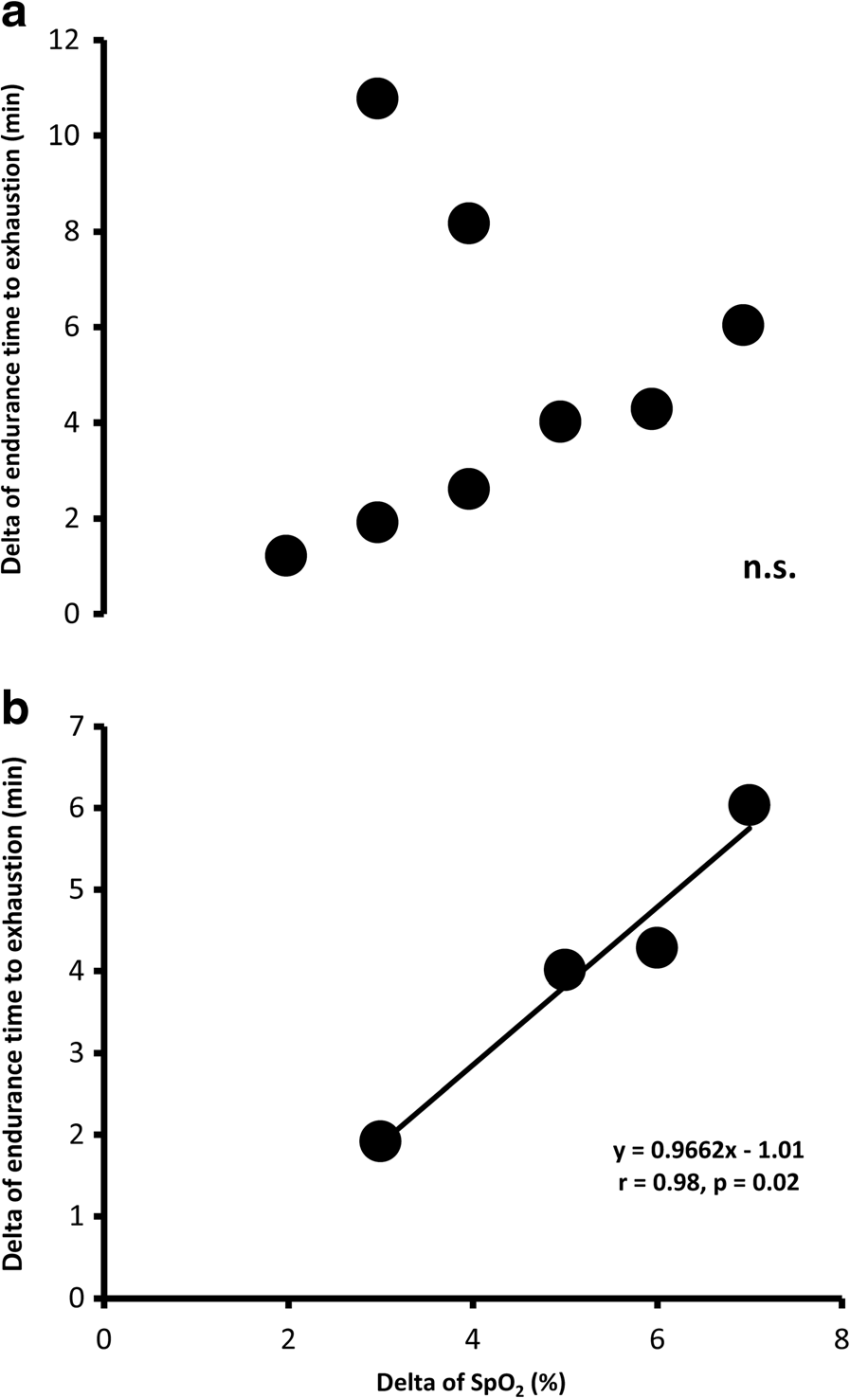
Correlations between the delta of SpO_2_ (the values between hyperoxia and normoxia at exhaustion) and the delta of endurance time to exhaustion (the values between hyperoxia and normoxia) were measured in **a** all participants and **b** participants who exhibited exercise-induced arterial hypoxemia during the intermittent cycling exercise tests under normoxic conditions

The individual endurance times to exhaustion during the intermittent cycling exercise tests are shown in Fig. 1. The endurance time to exhaustion under HO conditions was significantly longer than under NO conditions (34.9 ± 4.6 vs. 30.0 ± 2.5 min, *P* = 0.004, ES as Cohen’s *d* = 1.32). No correlation was detected between the delta of SpO_2_ (the values between hyperoxia and normoxia at exhaustion) and the delta of endurance time to exhaustion (the values between hyperoxia and normoxia) in the participants (*r* = 0.15, *P* = 0.72, Fig. 2); however, a strong correlation was detected in participants who exhibited exercise-induced arterial hypoxemia during the intermittent cycling exercise tests under normoxic conditions (*r* = 0.98, *P* = 0.02, Fig. 2).

#### Cardiorespiratory Measurements and Blood Lactate Concentrations

**Fig. 3:**
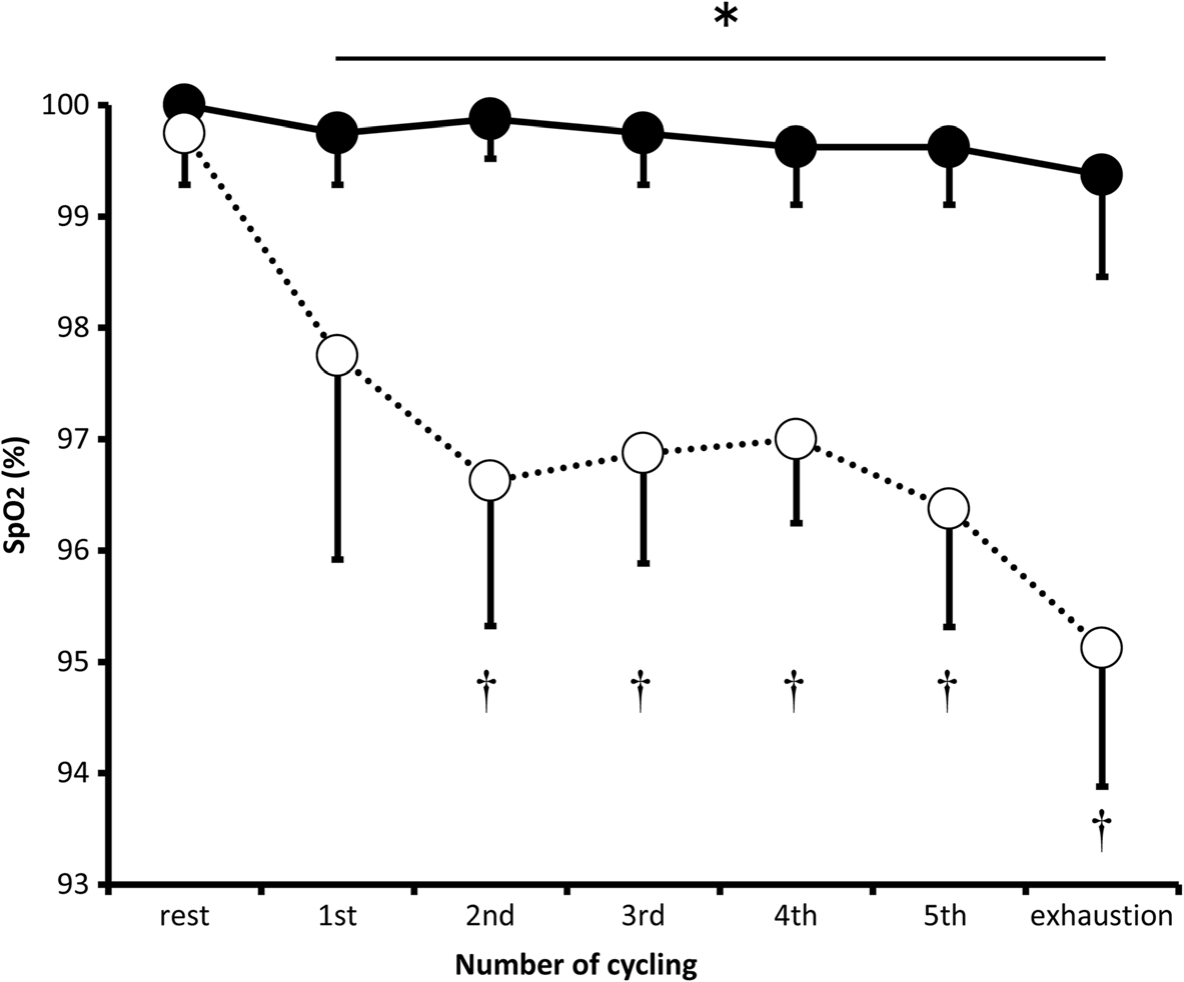
Mean arterial oxygen saturation (SpO_2_) levels were tested from 150 to 180 s during each high-intensity period. Hyperoxic (HO) conditions, FiO_2_ = 0.36, *black*; normoxic (NO) conditions, FiO_2_ = 0.21, *white*. Values were also averaged during the last 30 s of the fifth set of the tests (exhaustion). Values are means ± standard deviations (*n* = 8). *Significant difference vs. NO (*P* < 0.05). ^†^Significant difference vs. rest (*P* < 0.05)

The mean SpO_2_ values from 150 to 180 s during each 3 min high-intensity period of the intermittent cycling exercise test are shown in Fig. 3. The mean SpO_2_ values were significantly higher under HO conditions than under NO conditions during all sets, but not during rest periods (*P* = 0.018, 0.0001, 0.0001, 0.0001, 0.0001, and 0.0001 for sets one, two, three, four, five, and at exhaustion, respectively; *η*^2^ = 0.57, 0.88, 0.88, 0.88, 0.92, and 0.88, respectively). The mean SpO_2_ values during the second, third, fourth, and fifth sets and at exhaustion for the intermittent cycling exercise tests under NO conditions were significantly lower compared with those obtained during rest periods (*P* = 0.011, 0.004, 0.001, 0.001, and 0.0001, respectively; Cohen’s *d* = 3.19, 3.71, 4.39, 4.12, and 4.92, respectively). Table 1 shows the RPE during each set of the intermittent cycling exercise test, the mean HR from 150 to 180 s during each 3-min high-intensity cycling period, and the [La]_b_ at the end of each high-intensity cycling period. The RPE was significantly lower under HO conditions compared with NO conditions during sets one, three, and four (*P* = 0.008, 0.007, and 0.011, respectively; *η*^2^ = 0.66, 0.67, and 0.63, respectively). There was no significant difference in HR between HO and NO conditions. The [La]_b_ at the end of each high-intensity cycling period was lower under HO than NO conditions during all high-intensity cycling periods (*P* = 0.016, 0.007, 0.004, 0.003, and 0.002 during sets one, two, three, and four, and at exhaustion, respectively; *η*^2^ = 0.59, 0.67, 0.71, 0.73, and 0.78, respectively).

**Table 1:** Analysis of select parameters during intermittent cycling exercise tests under hyperoxia and normoxia

		1st	2nd	3rd	4th	5th	Exhaustion
RPE	Hyperoxia Normoxia	11 ± 2* 13 ± 2	12 ± 2 14 ± 2	13 ± 2* 15 ± 2	14 ± 3* 16 ± 2		18 ± 2 18 ± 2
HR (bpm)	Hyperoxia Normoxia	169 ± 7 172 ± 8	179 ± 8 181 ± 9	182 ± 9 183 ± 10	184 ± 8 185 ± 10	185 ± 8 187 ± 9	192 ± 9 192 ± 8
[La]_b_ (mmol l^−1^)	Hyperoxia Normoxia	6.9 ± 1.3* 9.1 ± 1.5	7.2 ± 1.5* 10.3 ± 1.9	6.8 ± 2.1* 10.8 ± 2.6	6.8 ± 2.2* 11.4 ± 2.9		9.7 ± 2.0* 14.0 ± 2.8

Values are expressed as means ± standard deviations

*Significant difference vs. normoxia (*P* < 0.05)

## Discussion

During the intermittent cycling exercise test under HO conditions the endurance time to exhaustion was significantly longer than under NO conditions. In addition, SpO_2_ was maintained under HO conditions, while it decreased under NO conditions. These findings support our hypothesis that inhalation of hyperoxic gas would improve high-intensity intermittent exercise performance and maintain S_a_O_2_.

All of the participants had longer endurance times to exhaustion under HO conditions than under NO conditions during high-intensity intermittent exercise (Fig. 1). Although there is evidence that exposure to hyperoxia improves performance during continuous exercise for more than 3 min [24, 27, 33], the effects of hyperoxic gas inhalation on performance during intermittent exercise have been controversial [30]. Exercise performance was improved by inhalation of hyperoxic gas during 6-min recovery periods between five repetitions of high-intensity bench swimming [30], but not by inhalation of hyperoxic gas during 2-min recovery periods between six 3-min laboratory-based kayak ergometer sessions [22]. Our current results demonstrate that inhalation of hyperoxic gas during five entire 3-min sessions of high-intensity intermittent exercise alternated with 3-min rest periods improves exercise performance.

High-intensity intermittent or continuous exercise causes a reduction in S_a_O_2_ [7, 21, 23]. A previous study demonstrated that S_a_O_2_ decreased from 95.0 to 88.7 % during high-intensity intermittent exercise [21]. Exercise-induced arterial hypoxemia occurs in approximately 50 % of athletes during continuous incremental exercise [25] and decreases in S_a_O_2_ which has negative effects on systemic O_2_ transport [13]. Exercise-induced arterial hypoxemia results in less oxygen availability to active muscles during maximal or near maximal exercise, which has a potentially detrimental effect on athletic performance. However, adaptations may exist in participants showing exercise-induced arterial hypoxemia [17] which means that performance may not be affected. The degree of influence of exercise-induced arterial hypoxemia on performance may differ depending on the participant’s particular adaptation.

No correlation between the delta of SpO_2_ (the values between hyperoxia and normoxia at exhaustion) and the delta of endurance time to exhaustion (the values between hyperoxia and normoxia) was detected in the participants; however, a strong correlation was detected in participants who exhibited exercise-induced arterial hypoxemia (*n* = 4) during the intermittent cycling exercise tests under normoxic conditions in this study. Exercise-induced arterial hypoxemia was defined as a progressive and persistent decrease in S_a_O_2_ during exercise and a drop of more than 4 % between resting levels and final levels after exercise had ceased [20, 26]. Grataloup et al. [11] reported that participants who exhibited exercise-induced arterial hypoxemia showed an improved $\overset{.}{\mathrm{V}}{\mathrm{O}}_{2 \max }$ compared with participants who did not exhibit exercise-induced arterial hypoxemia under hyperoxia conditions. Further studies are required to clarify the effect of hyperoxic gas inhalation on the relationship between participant characteristics and the magnitude of improved performance.

The SpO_2_ levels were maintained under HO but decreased under conditions of NO during the intermittent cycling exercise test in this study (Fig. 3). These findings are similar to those obtained by Nummela et al. [21], who reported that inhalation of hyperoxic gas (FiO_2_ = ~0.40) during exercise can completely prevent the fall in S_a_O_2_ that results from high-intensity intermittent exercise. When hyperoxic gas was inhaled during the recovery periods between high-intensity intermittent exercises, S_a_O_2_ increased from 95.8 to 99.8 % during the recovery periods but did not change under normoxic conditions [29]. In the current study, inhalation of hyperoxic gas during intermittent exercise prevented any decrease in S_a_O_2_.

Both ventilation/perfusion mismatching and diffusion limitation play a role in gas exchange alteration contributing to exercise-induced hypoxemia during high-intensity exercise [26]. Knight et al. [16] reported that hyperoxia (FiO_2_ = 1.00) increased leg $\overset{.}{\mathrm{V}}{\mathrm{O}}_{2 \max }$ and maximal O_2_ delivery during cycling exercise; this was due solely to the greater amount of oxygen in the blood [16, 34]. Inhalation of hyperoxic gas is also known to enhance the diffusion of O_2_ into mitochondria [16, 27, 28], which is one of the potential reasons for the improvement in performance observed under hyperoxic conditions.

The [La]_b_ at the end of each high-intensity cycling period was lower under HO than NO conditions (Table 1). This attenuation of [La]_b_ may have contributed to the enhanced intermittent exercise performance seen under HO conditions in our study [10, 18, 19]. Reduced [La]_b_ might also reflect a slower net breakdown of glycogen during the high-intensity cycling periods [18, 19, 32]. Attenuation of metabolic acidosis may slow the decline in muscle pH and delay inhibition of glycogen phosphorylase and phosphofructokinase [4, 31]; thus, the decrease in [La]_b_ appears to reflect deceleration of anaerobic energy production.

The RPE was lower under HO than NO conditions in this study. This is in agreement with the findings of Sperlich et al. [29], who reported that when cyclists were subjected to five 30-s high-intensity cycling intervals separated by 6 min of recovery, their RPE was lowered under HO conditions after the fourth and fifth intervals. The strong correlation between RPE and [La]_b_ at high workloads [23] suggests that one of the reasons for the lower RPE under HO conditions in this study was the lower [La]_b_ compared with that detected under NO conditions.

This study had several limitations. First, the number of participants was relatively small. However, the individual results clearly demonstrate that all participants had better endurance times to exhaustion during high-intensity intermittent exercise under HO than they did under NO conditions and these consistent findings are therefore likely to translate to a larger population.

Second, the homogenous profile of the participants was another limitation of the study; all were moderately trained male cyclists (mean VO_2peak_ 59.1 mL kg^−1^ min^−1^), and no elite athletes were included in this study. Hopkins et al. [15] reported that moderately trained female participants developed exercise-induced arterial hypoxemia during maximal treadmill running and that their arterial oxygen values did not differ from those previously measured in men. Several studies have suggested that elite endurance athletes may experience reductions in S_a_O_2_ and exercise hypoxemia during maximal or submaximal exercise, but great individual variation in the S_a_O_2_ reduction has been reported, especially in elite athletes, with reported $\overset{.}{\mathrm{V}}{\mathrm{O}}_{2 \max }$ values of 72 mL kg^−1^ min^−1^ [6] and 71.3 mL kg^−1^ min^−1^ [23]. Further studies are required to clarify the effect of hyperoxic gas inhalation on the relationship between the physical fitness level and exercise performance.

## Conclusions

Hyperoxic gas inhalation during the entire high-intensity intermittent exercise test enhanced exercise performance in male cyclists. Further studies are required to clarify whether high-intensity intermittent exercise training with hyperoxic gas inhalation is more beneficial than with normoxic gas.

## Abbreviations

FiO_2_: Fraction of inspiratory oxygen
HO: Hyperoxia
HR: Heart rate
[La]_b_: Blood lactate concentration
NO: Normoxia
RPE: Rating of perceived exertion
S_a_O_2_: Hemoglobin oxygen saturation
SpO_2_: Arterial oxygen saturation
$\overset{.}{\mathrm{V}}{\mathrm{O}}_{2\mathrm{peak}}$: Peak oxygen uptake
